# Agriculture

**Published:** 1859-11

**Authors:** 


### AGRICULTURE.

Nine times out of ten the best answer which a physician can give to the patient, who, with direful look and dolorous tone, inquires what shall I do? is, *go to work.*

The most important injunction that can be given to this fast age, whether in regard to solid financial prosperity, or to enduring personal enjoyment, or to gladness of heart, or health of body, is, be content with a slow and moderate increase of your substance.

The crying educational error of the age is, allowing so many boys and girls to reach adult life without the knowledge of some handicraft, by which they might earn a living in any country, in case they were reduced to penury. There are scores of thousands of persons in this country who are living from hand to mouth, whose loss of a single day's labour would be followed by a dinnerless day, who might live in careless comfort on a single acre of land, but for the want of a little patient industry and self denial. Look at it:

A single acre of land will readily afford room for forty apple trees, and forty bushels to a tree is not an uncommon product, making sixteen hundred bushels of fruit, which, in mid-winter in any of our large cities or large towns, will readily bring, if in good order, half a dollar a bushel, and sometimes a dollar, by the barrel. A plain, industrious, and econimical family in the country can live comfortably on half that amount of money.

Nicholas Longworth, of Cincinnati, to whose industry, sagacity, and enterprise this nation owes a large debt for what he has done to promote the culture and perfection of the strawberry and the grape, writes, that in Germany an acre of grapes will yield eight hundred gallons of wine, whose lowest value is one dollar, in its state, and that the same yield can be had here, and when once in bearing, one-half is clear profit.

A New England farmer, of forty years' experience, writes that he raises six hundred bushels of onions on an acre of land; that at the last weeding in August he sows turnip seed, and gathers a crop of four hundred bushels; each of these sell in New York, and other large cities and towns, and sell readily by wholesale, for eighty cents a bushel, in almost any year.

An acre of cold, marshy, sandy land will yield forty barrels of cranberries, which often sell for thirty dollars a barrel.

An acre of the common white bean, which is easily cultivated, requires but little skill, and which is not affected by frost or rot, and which is always a saleable article, will yield an equally profitable crop, if well managed.

J. W. Manning says he cultivated a piece of ground "on which was an orchard of apple trees, some of them four inches in diameter; one hundred and fifty grape vines, part of them in bearing; a hundred and thirty current bushes in bearing; fifty hills of rhubarb, and one-third of the whole in the Cutter strawberry, which, in a season of thirty-five days, yielded five hundred quarts. And all on one-fifth of an acre of ground!"

With these facts before us, we say to all, if you want to live long in health and quiet and independence, go to work in the love of it, be satisfied with moderate gains, cultivate moderate ambitions, practice self-denials, and you will reap a rich reward here and hereafter.

